# PReS-FINAL-1011: Can repeated T cell receptor stimulation lead to epigenetic reprogramming of the treg-specific demethylated region in human conventional T cells?

**DOI:** 10.1186/1546-0096-11-S2-P9

**Published:** 2013-12-05

**Authors:** Q Wu, D Bending, LR Wedderburn

**Affiliations:** 1 Imperial College London, London, UK; 2 Rheumatology Unit, Institute of Child Health, University College, London, UK

## Introduction

Regulatory T (Treg) cells, vital to prevent autoimmune disease, can be identified by their expression of the forkhead box P3 (FoxP3) transcription factor. Human conventional T (Tconv) cells stimulated via the T cell receptor (TCR) can also express FoxP3. Although this can confer some intrinsic regulatory effects, controversy exists over whether FoxP3 expression alone gives rise to the Treg cell phenotype. Treg-specific demethylated region (TSDR) demethylation is thought to be a reliable marker of commitment to the Treg cell lineage. In most human studies, analysis of TSDR methylation status has been performed on bulk populations, where only a subpopulation of cells express FoxP3. However, TSDR demethylation may occur selectively in cells expressing the highest levels of FoxP3 protein. Previously, investigation of epigenetic modifications in FoxP3^+ ^human Tconv cells has been hampered by the inability to separate cells on the basis of FoxP3 expression. Recently, however, a protocol has been published detailing a method for DNA extraction from cells that have been fixed and stained for FoxP3, permitting more informative phenotyping of TSDR methylation status.

## Objectives

To examine the kinetics and stability of FoxP3 expression in human Tconv cells undergoing repeated TCR stimulation; in addition to analyze the TSDR methylation status on cells separated based on FoxP3 expression.

## Methods

Cells were separated into CD4^+^CD25^hi^CD127^lo ^(Treg) and CD4^+^CD25^-^CD127^hi ^(Tconv) populations and cultured for 3 weeks with anti-CD3, anti-CD28, cytokine combinations and, in some experiments, demethylating agent 5-azacytidine (5-azaC). At regular intervals, cells were analyzed for expression of Treg cell markers. On days 7 and 16, Tconv cells were sorted into CD4^+^CD25^+^FoxP3^+ ^and CD4^+^CD25^+^FoxP3^- ^populations for DNA extraction and bisulfite sequencing to analyze TSDR methylation status.

## Results

Activation-induced FoxP3 expression in Tconv cells was augmented by interleukin-2 (IL-2), but was unstable. TSDR became partially demethylated in the day 7 FoxP3^+ ^population in one of two donors. 5-azaC stabilized FoxP3 protein expression and this was associated with a small increase in overall TSDR demethylation.

## Conclusion

FoxP3 protein expression alone may not be an adequate marker of Treg cells in states of chronic stimulation, where a notable proportion of FoxP3-expressing cells may be recently activated Tconv cells. TSDR demethylation may be a more specific marker of commitment to the Treg cell lineage. However, preliminary results suggest a small subpopulation of FoxP3-expressing Tconv cells may demethylate at the TSDR in response to TCR stimulation, warranting further investigation. This work may contribute towards understanding how induced Treg cells could be stably generated *in vitro, *with potential applications in adoptive transfer therapies for the treatment of autoimmune disease.

## Disclosure of interest

None declared.

